# Air pollution exposure is associated with gene expression in children

**DOI:** 10.1093/eep/dvae025

**Published:** 2024-12-21

**Authors:** Siddhartha Das, Amanda Rundblad, Irene Fontes Marques, Ana Goncalves Soares, Vincent W Jaddoe, Martine Vrijheid, Mark Nieuwenhuijsen, Joost Verlouw, Jason Matthews, Kirsten B Holven, Magne Thoresen, Nicholas J Timpson, Janine F Felix, Stine M Ulven

**Affiliations:** Department of Nutrition, Institute of Basic Medical Sciences, University of Oslo, Oslo 0317, Norway; Department of Nutrition, Institute of Basic Medical Sciences, University of Oslo, Oslo 0317, Norway; Generation R Study Group, Erasmus MC, University Medical Center Rotterdam, Rotterdam 3015 GD, the Netherlands; Department of Pediatrics, Erasmus MC, University Medical Center Rotterdam, Rotterdam 3015 GD, The Netherlands; MRC Integrative Epidemiology Unit at the University of Bristol, Bristol BS8 2BN, United Kingdom; Population Health Sciences, Bristol Medical School, University of Bristol, Bristol BS8 2BN, United Kingdom; Generation R Study Group, Erasmus MC, University Medical Center Rotterdam, Rotterdam 3015 GD, the Netherlands; Department of Pediatrics, Erasmus MC, University Medical Center Rotterdam, Rotterdam 3015 GD, The Netherlands; Institute for Global Health Barcelona, Dr. Aiguader 88, Barcelona 08003, Spain; Institute for Global Health Department of Experimental and Health Sciences, Pompeu Fabra University, Barcelona 08002, Spain; CIBER de Epidemiología y Salud Pública (CIBERESP), Madrid 28029, Spain; Institute for Global Health Barcelona, Dr. Aiguader 88, Barcelona 08003, Spain; Institute for Global Health Department of Experimental and Health Sciences, Pompeu Fabra University, Barcelona 08002, Spain; CIBER de Epidemiología y Salud Pública (CIBERESP), Madrid 28029, Spain; Generation R Study Group, Erasmus MC, University Medical Center Rotterdam, Rotterdam 3015 GD, the Netherlands; Department of Pediatrics, Erasmus MC, University Medical Center Rotterdam, Rotterdam 3015 GD, The Netherlands; Department of Pharmacology and Toxicology, University of Toronto, 1 King’s College Circle, Toronto, ON M5S 1A8, Canada; National Advisory Unit on Familial Hypercholesterolemia, Department of Endocrinology, Morbid Obesity and Preventive Medicine, Oslo University Hospital, Oslo 0424, Norway; Department of Biostatistics, Institute of Basic Medical Sciences, University of Oslo, Oslo 0317, Norway; MRC Integrative Epidemiology Unit at the University of Bristol, Bristol BS8 2BN, United Kingdom; Population Health Sciences, Bristol Medical School, University of Bristol, Bristol BS8 2BN, United Kingdom; Generation R Study Group, Erasmus MC, University Medical Center Rotterdam, Rotterdam 3015 GD, the Netherlands; Department of Pediatrics, Erasmus MC, University Medical Center Rotterdam, Rotterdam 3015 GD, The Netherlands; Department of Nutrition, Institute of Basic Medical Sciences, University of Oslo, Oslo 0317, Norway

**Keywords:** ALSPAC, Generation R, gene expression, interferon, natural spaces, PM_2.5_

## Abstract

Environmental exposures, including air pollutants and lack of natural spaces, are associated with suboptimal health outcomes in children. We aimed to study the associations between environmental exposures and gene expression in children. Associations of exposure to particulate matter (PM) with diameter <2.5 (PM_2.5_) and < 10 (PM_10_) micrometers, nitrogen dioxide, green spaces, and blue space, with whole-blood gene expression were explored in children from the Dutch Generation R Study (*n* = 172). Analyses were adjusted for age, sex, batch, maternal education, and area socioeconomic status. Follow-up analysis was carried out using lymphoblastoid cell line gene expression in children from the ALSPAC Study (*n* = 946). Gene set enrichment analysis (GSEA) using hallmark and immune gene sets from the molecular signature database was carried out to identify significantly over-represented gene sets for insights into biological mechanisms Exposure to PM_2.5_ was associated with expression of 86 genes in discovery analyses in the Generation R Study [false discovery rate (FDR)-adjusted *P*-value < .25]. Of these, PM_2.5_ was also associated with *GNG11* expression in the same direction in follow-up analysis (FDR-adjusted *P*-value < .05). The remaining exposures showed much fewer associations in the discovery analyses. GSEA using PM_2.5_ association results for both cohorts indicated suppression of gene sets related to interferon response and response to bacterial and viral exposure. In conclusion, gene expression analysis performed in two independent cohorts suggests that PM_2.5_ exposure in children may be involved in interferon and microbial infection responses.

## Graphical Abstract

**Figure:**
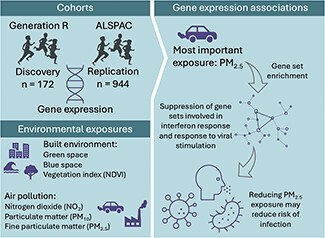

## Introduction

Air pollution is estimated to account for 6.7 million global deaths and 45% of these are from cardiovascular disease (CVD) [1–3]. Recent data show that particulate matter (PM) air pollution is the leading risk factor of global disease burden [4]. Additionally, air pollution is among the major contributors to health burden in children aged ∼9 years [2]. Exposure to air pollutants in infancy is also reported to adversely affect CVD risk factors such as increased levels of low-density lipoprotein levels [5] and blood pressure [6]. CVD is an important burden of disease in youths and young adults [7]. It is therefore important to understand the adverse effects of air pollution to minimize CVD risk not only in adults but also in children.

Mechanistically, air pollutants can stimulate cells through a variety of cellular sensing mechanisms including toll-like receptors (TLRs), reactive oxygen species (ROS), and poly-aromatic hydrocarbon (PAH) sensing pathways causing inflammation and oxidative stress [8–10]. These pathways can be initially activated in the airways and lungs, and the release of biological intermediates can then reach the blood circulation and affect the risk of CVD [3]. Human studies are needed to find molecular targets of air pollutant exposure to devise better preventive measures against the adverse effects of air pollution, especially in early life.

In adults, several cohorts have found positive and negative associations of PM < 2.5 µm (PM_2.5_) exposure and whole-blood DNA methylation at CpG sites mapped to genes related to CVD risk such as immunity and inflammation [11–14] as well as lung function [15]. Daily PM < 10 µm (PM_10_) exposure has also been shown to be inversely associated with DNA methylation of inflammatory genes, measured in peripheral blood of adults with overweight/obesity [16]. In children, PM_2.5_ exposure is associated with altered DNA methylation of genes involved in immune regulation in peripheral blood mononuclear cells (PBMCs) [17]. In newborns, PM_2.5_ exposure has been associated with altered DNA methylation levels [18, 19], and recent epigenome-wide meta-analysis showed that the prenatal PM exposure was associated with altered DNA methylation in or near genes relevant for respiratory health and lung function [20]. Another pooled epigenome-wide association study showed that exposure to air pollutants during pregnancy was associated with placental DNA methylation near genes involved in immunity and metabolism [21]. Nitrogen dioxide (NO_2_) exposure during pregnancy has also been shown to be associated with DNA methylation in genes involved in mitochondrial function, inflammation, and antioxidant defense pathways in newborn [22].

Since altered DNA methylation levels can affect gene expression, some studies have addressed associations of air pollutants on peripheral blood gene expression in adults [23, 24]. PM_2.5_ exposure is associated with expression of genes involved in cell signaling and immune response [24], while NO_2_ exposure is positively associated with expression of inflammatory genes such as interleukin-8 (IL-8) in PBMCs [23]. Few studies have used a whole-transcriptome approach to study the associations between air pollutants and gene expression levels in children [25, 26]. One study compared gene expression levels in high-polluted area with a low-polluted area and found that the main biological processes associated with air pollution exposure were nucleosome, chromatin assembly, nucleic acid metabolism, and RNA splicing [25]. A meta-analysis of transcriptome-wide association studies in children and adolescents from Europe found that exposure to PM_2.5_ was associated with two differentially expressed transcript clusters at birth [26].

Green space, a measure of surrounding plants and vegetation, has been associated with lower mortality and beneficial physical and mental health effects over the life course [27–29]. Green space exposure is also reported to reduce the harmful effects of air pollutants by absorbing and filtering PM [30]. A few studies have investigated the associations between green space exposure and blood DNA methylation in children and adults as well as cord blood in newborns [31–33]. Growing evidence also suggests that spending time in or around water bodies or ‘blue spaces’ is associated with mental well-being [34], but it is unknown if this environmental factor is associated with gene expression or DNA methylation.

The LongITools consortium has brought together multiple longitudinal studies with extensive data on various environmental exposures including air pollutants and green and blue spaces to study the effect of environmental exposures on human health and disease [35]. Differential gene expression may underlie some of the observed associations of air pollution and natural spaces with health outcomes. To examine this, the objective of this exploratory study was to investigate the association between environmental exposures and gene expression in children around 10 years of age using two cohorts from this consortium, the Generation R Study, in the Netherlands, and the Avon Longitudinal Study of Parents and Children (ALSPAC), in the UK. These two have whole-transcriptome data from whole blood and lymphoblastoid cell lines, respectively, and where the only cohorts with childhood gene expression data in LongITools.

## Results

### Population and covariate characteristics

Final gene expression analyses were performed on data from 172 children in the Generation R Study and 946 children from the ALSPAC Study. In both populations, about 47% of the participants were boys and the mean age of the participants was around 9.8 years (Table 1). In the Generation R Study, 72.7% of the participants with gene expression data had mothers with higher education (college and higher), while 26.1% had completed some form of secondary education. In the ALSPAC cohort with gene expression data, 82.5% of the participants had mothers who had some form of post-secondary education. In the Generation R Study, 58.7% of the participants were from the top two area socioeconomic strata with no participants from the bottom two strata, while in ALSPAC, 44.3% of the participants were from the top two strata and 24.2% of the participants were from the bottom two strata. For the air pollutant exposure distributions, there was higher average exposure to PM_2.5_ and NO_2_ in Generation R compared with ALSPAC, but there was a slightly higher average exposure to PM_10_ in the ALSPAC cohort. Most of the Generation R participants (73.3%) lived within 300 m of a green space as well had dense vegetation from 100 to 500 m of their residency as indicated by the normalized difference vegetative index (NDVI) values. For blue space exposure, 52.3% of the Generation R participants lived within 300 m of a water body.

**Table 1.** Summary of the exposures and covariates for samples in Generation R and ALSPAC used for association with gene expression

	Unit/category	Generation R (n = 172)	ALSPAC (n = 946)
*Child sex, n (%)*	Male	80 (46.5)	449 (47.5)
*Child age, mean (SD)*	Years ± SD	9.84 ± 0.3	9.8 ± 0.3
*Mothers’ education, n (%)*	No higher education	45 (26.1)	98 (CSE, 10.5) 66 (Vocational, 7)
	Higher education	125 (72.7)	333 (O level, 35.6) 263 (A level, 28.1) 175 (Degree, 18.7)
	Missing	2 (1.2)	11 (1.2)
*Area SES, n (%)*	1 (highest)	76 (44.2)	243 (25.7)
	2	25 (14.5)	176 (18.6)
	3	68 (39.5)	189 (20)
	4	N/A	128 (13.5)
	5	N/A	101 (10.7)
	Missing	3 (1.8)	109 (11.5)
*PM_2.5_, µg/m^3^*	Mean ± SD	13.8 ± 0.95	10.99 ± 0.7
	Maximum	17.6	13.3
	25th Percentile	13.2	10.6
	75th Percentile	14.3	11.4
	Missing, *n*	0 (0 %)	109 (11.5%)
*PM_10_ µg/m^3^*	Mean ± SD	21.7 ± 1.97	23.31 ± 1.15
	Maximum	30.5	26.8
	25th Percentile	20.3	22.3
	75th Percentile	22.7	24
	Missing, *n*	0 (0%)	149 (15.8%)
*NO_2_, µg/m^3^*	Mean ± SD	28.6 ± 8.02	22.2 ± 3.55
	Maximum	103	30.2
	25th Percentile	25	19.8
	75th Percentile	31.2	24.5
	Missing, *n*	0 (0%)	109 (11.5%)
*Green space, n (%)*	Within 300 m	126 (73.3)	
	Missing	23 (13.4)	
*NDVI, within 100 m*	Mean (SD)	0.44 (0.11)	
	Maximum	0.8	
	25th Percentile	0.4	
	75th Percentile	0.5	
*NDVI, within 300 m*	Mean (SD)	0.5 (0.09)	
	Maximum	0.8	
	25th Percentile	0.4	
	75th Percentile	0.5	
*NDVI, within 500 m*	Mean (SD)	0.5 (0.08)	
	Maximum	0.8	
	25th Percentile	0.4	
	75th Percentile	0.5	
*Blue space, n (%)*	Within 300 m	78 (52.3)	
	Missing	23 (13.4)	

To check if the sub-cohort chosen for gene expression analyses biases the analysis results, we also looked at the summary statistics for the same covariates and exposures in the whole cohort. For both studies, sex and age distributions were very similar for the included and the non-included populations (Supplementary Table S1). There were some differences in maternal educational level, with included participants in Generation R having a lower percentage of highly educated mothers as compared to the non-included participants, and ALSPAC showing the opposite. In both studies, the included population had a larger percentage of participants from neighborhoods with higher area socioeconomic status (SES). Exposure levels to air pollutants were largely similar between included and non-included participants, and the percentage with a large green space within 300 m of their home was somewhat lower in the included population in Generation R. Overall, most of the variables in the sub-cohort chosen for the gene expression analyses are similar to the whole cohort.

### Association between exposures and gene expression in generation R

**Figure 1.**
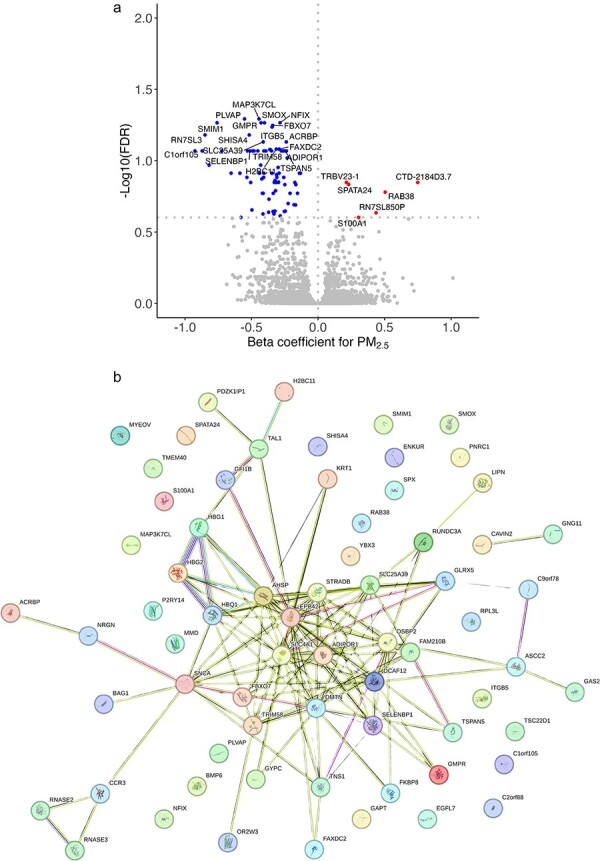
Summary of associations between PM_2.5_ exposure and gene expression adjusted for age, sex, batch, maternal education, and SES for Model 1 in the discovery sample. Volcano plot for PM_2._exposure and association on gene expression (red—positive association, blue—negative association) (a), STRING protein–protein interaction map for 67 mapped PM_2.5_ associated genes (b).

Among the 18 787 genes chosen for the final analysis, PM_2.5_ exposure was associated with expression of 86 genes after false discovery rate (FDR) correction (*P* < .25) of which 80 (93%) were negatively associated (Supplementary Table S2, Fig. 1a). For these 86 genes, we found that there were 132 known edges of the 69 annotated proteins that could be mapped to the STRING database as compared to an expected number of nine with an enrichment *P*-value for interactions of <1.0e-16 (Fig. 1b). This suggests that there are known and predicted physical and functional interactions of the corresponding protein products of these genes.

**Figure 2.**
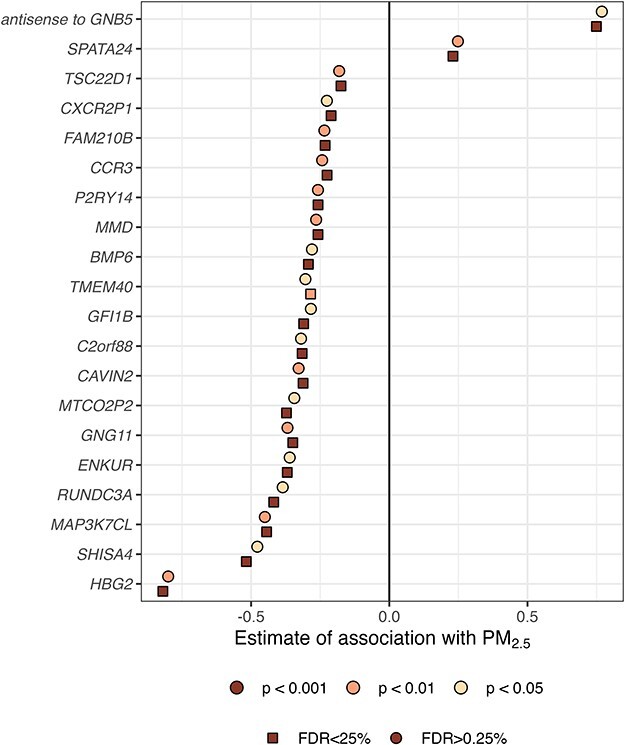
Forest plot for regression coefficients of 20 genes of which expression was associated with PM_2.5_ in Generation R adjusted for age, sex, batch, maternal education, and area SES in Model 1 and that were also significant at an unadjusted *P*-value < .01 in Model 2, which was additionally adjusted for cell-type composition. For each gene, the square box corresponds to PM_2.5_ coefficient estimate for a gene in Model 1 while circle corresponds to estimate in Model 2. The color within the box or circle corresponds to unadjusted *P*-value estimate in Model 2.

Among the 86 expressed genes showing association with PM_2.5_ in Model 1, none were associated with PM_2.5_ in Model 2 after adjustment for cell-type composition (Supplementary Table S3) and FDR correction at adjusted *P*-value < .25. This may have been due to only 121 samples having cell-type composition data for analysis in Model 2, and thus we wanted to find genes in Model 1 for which the association did not seem to be strongly confounded by cell-type differences. We focused on those genes which showed <10% change in the regression coefficient from Model 1 to Model 2 and was significant at a nominal *P*-value level of .05 and found 20 such genes out of the 86 (Table 2 and Fig. 2).

**Table 2.** PM_2.5_ associated genes in Generation R (FDR-adjusted *P*-value < .25) in Model 1 that had a beta coefficient difference of <10% in Model 2^a^

Gene symbol	Beta estimate in Model 1	Nominal *P*-value in Model 1	FDR-adjusted *P*-value in model 1	Beta estimate Model 2	Nominal *P*-value in Model 2	Change in log_2_ fold change in Model 2 relative to Model 1 (%)
*HBG2*	−0.819	.0002	.11	−.799	.009	2.35
*SHISA4*	−0.517	.00003	.07	−0.477	.010	7.69
*MAP3K7CL*	−0.444	.00000424	.11	−0.450		1.44
*RUNDC3A*	−0.418	.0006	.17	−0.386	.023	7.81
*MTCO2P2*	−0.372	.0009	.23	−0.344	.047	7.62
*ENKUR*	−0.369	.0007	.19	−0.360	.029	2.39
*GNG11*	−0.349	.0001	.09	−0.368	.002	5.50
*C2orf88*	−0.315	.00045	.14	−0.319	.015	1.27
*CAVIN2*	−0.312	.0002	.13	−0.328	.006	5.18
*GFI1B*	−0.310	.0003	.13	−0.284	.018	8.43
*BMP6*	−0.293	.0002	.12	−0.280	.017	4.39
*TMEM40*	−0.285	.001	.2	−0.303	.019	6.62
*MMD*	−0.258	.0003	.13	−0.265	.01	2.60
*P2RY14*	−0.257	.0003	.13	−0.258	.007	0.12
*FAM210B*	−0.232	.00016	.10	−0.235	.009	1.17
*CCR3*	−0.225	.0007	.2	−0.243	.005	7.92
*CXCR2P1*	−0.211	.0006	.17	−0.226		7.26
*TSC22D1*	−0.175	.00043	.14	−0.181	.009	3.73
*SPATA24*	0.230	.005	.18	0.248	.006	7.79
*Lnc-MAPK6-6 (long non coding RNA)/Novel transcript antisense to GNB5*	0.749	.01	.14	0.769	.011	2.63

a EdgeR does not produce standard errors and hence no estimates are included in this table.

For the remaining exposures, green space exposure was associated with expression of seven genes in Model 1, while NDVI at 100 and 300 m and PM_10_ exposures were associated with expression of one gene each (FDR < 0.25) (Table 3, Supplementary Table S2). Of the seven genes associated with green space, six were negatively associated (Table 4). The remaining exposures were not associated with gene expression.

**Table 3.** Summary of the number of environmental exposures associated genes in Generation R in Model 1

	Number of significant expressed genes (FDR-adj. *P* < .25)	
Exposure	Positive	Negative
PM_2.5_	6	80
Green space, within 300 m	1	6
NDVI, 100 m	1	0
NDVI, 300 m	1	0
PM_10_	0	1
NDVI, 500 m	0	0
Blue space, within 300 m	0	0
NO_2_	0	0

**Table 4.** List of all environmental exposures associated genes other than PM_2.5_ in Model 1^a^

Exposure	Beta estimate	Nominal *P*-value in Model 1	FDR-adjusted *P*-value in Model 1	Gene name
Green space, within 300 m	−2.265	4.72e-13	8.86e-09	*IGKV2-30*
Green space, within 300 m	−2.064	5.86e-10	5.51e-06	*BTN1A1*
Green space, within 300 m	−1.631	2.82e-9	1.77e-05	*IGKV2D-30*
Green space, within 300 m	−1.279	3.77e-06	0.01	*IGKJ5*
Green space, within 300 m	−1.255	1.06e-05	0.04	*IGHV1-18*
Green space, within 300 m	−1.188	7.47E-05	0.20	*IGHA1*
NDVI, 100 m	−0.996	3.72e-06	0.07	*MST1R*
NDVI, 300 m	−0.996	8.54e-07	0.02	*MST1R*
PM_10_	−0.347	2.089678e-06	0.043925878	*IGLV1-41*
Green space, within 300 m	0.808	6.41e-05	0.20	*MYO5C*

a EdgeR does not produce standard errors and hence no estimates are included in this table.

### Gene ontology enrichment analysis for the PM_2.5_-associated genes

**Figure 3.**
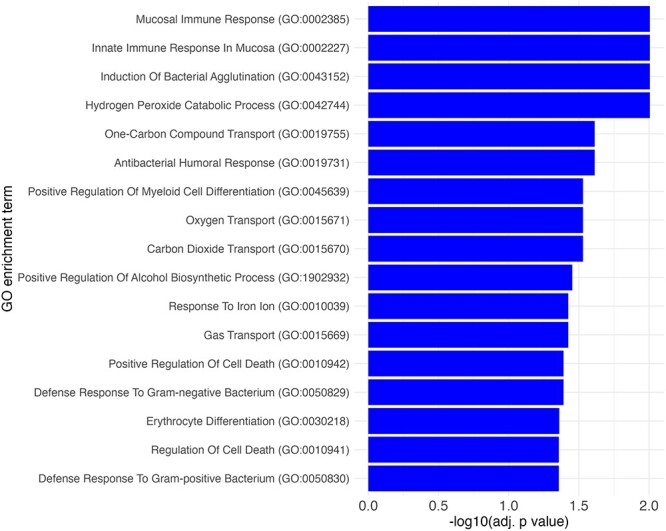
GO Biological process (BP) significantly enriched terms barplot for 86 genes showing suggestive association with PM_2.5_ in Model 1 ranked by most significant term to least significant term sorted by adjusted *P*-value. X axis lists the −log10 of adjusted *P*-value for the GO term and Y axis carries the name of the GO term.

We carried out the gene ontology (GO) enrichment analysis only for the PM_2.5_-associated genes before cell-type correction (*n* = 86) (Supplementary Table S2). GO enrichment analysis showed enrichment for mucosal innate immune response genes such as *H2BC11, RNASE2*, and *RNASE3*. These genes were also enriched for terms such as antibacterial humoral response and response to Gram-negative and -positive bacteria (Fig. 3, Table 5). There was also enrichment for gas transport terms including carbon dioxide and oxygen transport, which included the genes *HBG2* and *HBG1*. The enrichment terms positive regulation of alcohol biosynthesis and response to iron ion both shared the genes *BMP6* and *SNCA*. The genes *HBG1* and *HBG2* along with *SNCA* were also significantly enriched in the terms related to cell death.

**Table 5.** Significantly enriched GO terms for PM_2.5_ exposure-associated genes (*n* = 86)

Term	FDR-Adj. *P*-value	Genes
Innate immune response in mucosa (GO:0002227)	.009	*H2BC11;RNASE3;RNASE2*
Mucosal immune response (GO:0002385)	0.009	*H2BC11;RNASE3;RNASE2*
Induction of bacterial agglutination (GO:0043152)	0.009	*RNASE3;RNASE2*
Hydrogen peroxide catabolic process (GO:0042744)	0.009	*HBG2;HBG1;HBQ1*
Antibacterial humoral response (GO:0019731)	0.024	*H2BC11;RNASE3;RNASE2*
One-carbon compound transport (GO:0019755)	0.024	*HBG2;HBG1;SLC4A1*
Positive regulation of myeloid cell differentiation (GO:0045639)	0.029	*FAM210B; TAL1;FAXDC2*
Carbon dioxide transport (GO:0015670)	0.029	*HBG2;HBG1*
Oxygen transport (GO:0015671)	0.029	*HBG2;HBG1*
Positive regulation of alcohol biosynthetic process (GO:1 902 932)	0.035	*BMP6;SNCA*
Gas transport (GO:0015669)	0.038	*HBG2;HBG1*
Response to iron ion (GO:0010039)	0.038	*BMP6;SNCA*
Defense response to Gram-negative bacterium (GO:0050829)	0.041	*H2BC11;RNASE3;RNASE2*
Positive regulation of cell death (GO:0010942)	0.041	*HBG2;HBG1;SNCA*
Erythrocyte differentiation (GO:0030218)	0.044	*DMTN; TAL1;AHSP*
Defense response to Gram-positive bacterium (GO:0050830)	0.044	*H2BC11;RNASE3;RNASE2*
Regulation of cell death (GO:0010941)	0.044	*HBG2;HBG1;SNCA*

### Follow-up of PM_2.5_-associated genes in ALSPAC cohort

PM_2.5_ exposure was associated with 30 probes (FDR < 0.05) in the follow-up analysis in the ALSPAC cohort, and among these, PM_2.5_ exposure was positively associated with five probes (17%) and negatively associated with 25 probes (83%). For follow-up of the Generation R results in ALSPAC, we subset the results to only the probes corresponding to the 86 expressed genes that were associated with PM_2.5_ exposure in Generation R, and then adjusted for multiple testing correction. Forty of these genes mapped to a probe in the ALSPAC dataset, and among these, PM_2.5_ exposure was positively and negatively associated with expression of only two genes, *GYPC* and *GNG11*, of which *GNG11* showed the same direction of association as in Generation R (Table 6).

**Table 6.** PM_2.5_ associated genes from Generation R followed up in ALSPAC

Probe	Gene symbol	Beta estimate	*P*-value	FDR-adj. *P*-value
ILMN_1782419	*GNG11*	−0.06	.00014951	.00351339

### Gene set enrichment analysis for PM_2.5_ association results in Generation R and ALSPAC using hallmark and immune gene sets

**Figure 4.**
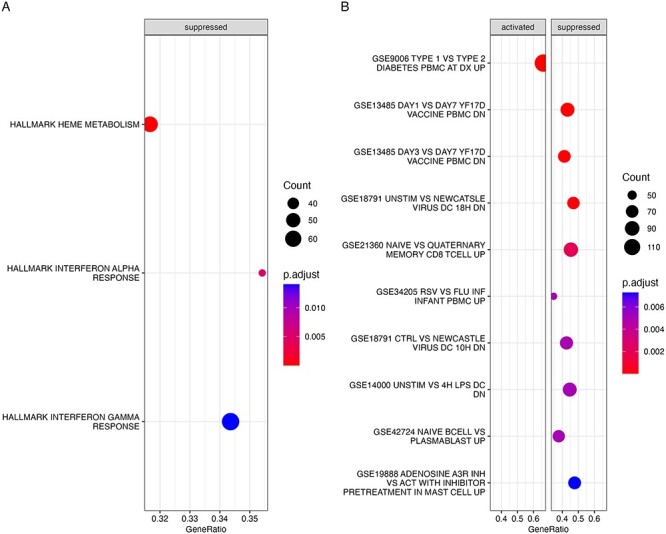
GSEA using PM_2.5_ gene expression association analyses for the Generation R cohort adjusted for cell type ordered by adjusted *P*-value. All the significant gene sets ranked by adjusted *P*-value from GSEA are displayed from top to bottom for hallmark gene sets (a) and the top 10 most significant ones for the immunologic gene sets (b). The names of the gene sets starting with GSE or Hallmark are listed on the left of each plot. The size of the circle for each gene set corresponds to the number of genes from the gene set that were present in association analysis results and the color corresponds to the FDR-adjusted *P*-value from the GSEA result with red indicating more significant results. The GeneRatio is a fractional measure of how many genes from the corresponding gene set were present in the current analysis. “suppressed” indicates that these gene sets had a negative enrichment score.

**Figure 5.**
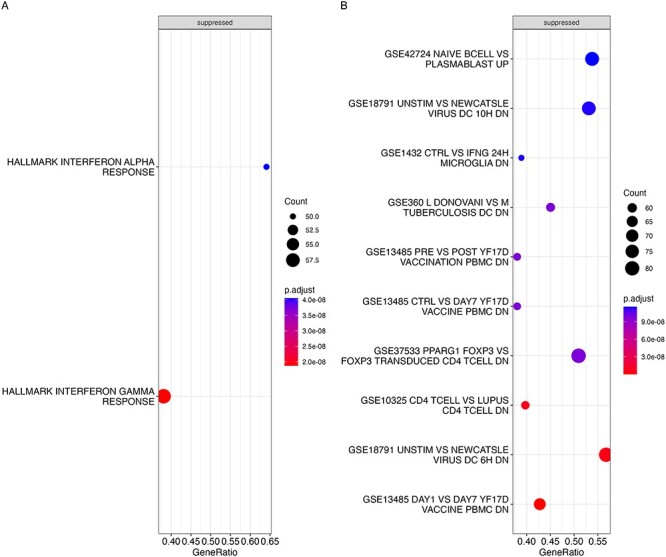
GSEA using PM_2.5_ gene expression association analyses for the ALSPAC cohort are ordered by adjusted *P*-value. All the significant gene sets ranked by adjusted *P*-value from GSEA or Hallmark are displayed from top to bottom for hallmark gene sets (a) and the top 10 most significant ones for the immunologic gene sets (b). The names of the gene sets starting with GSE are listed on the left of each plot. The size of the circle for each gene set corresponds to the number of genes from the gene set that were present in association analysis results and the color corresponds to the FDR-adjusted *P*-value from the GSEA result with red indicating more significant results. The GeneRatio is a fractional measure of how many genes from the corresponding gene set were present in the current analysis. “Suppressed” indicates that these gene sets had a negative enrichment score.

Gene set enrichment analysis (GSEA) using the hallmark gene sets for both the Generation R and ALSPAC cohorts showed statistically significant normalized negative enrichment scores after FDR correction for gene sets involved in interferon alpha and gamma response (Figs 4a and 5a). The interferon alpha and gamma response pathways are involved in response to external pathogens such as viral and bacterial infection. To gain further insights into immunological response regulated by PM_2.5_ exposure, we also carried out GSEA using the MSigDB immunologic gene sets and found that there were 39 and 414 such gene sets that showed statistically significant normalized enrichment scores after FDR correction at an adjusted *P*-value < .01 for Generation R and ALSPAC, respectively (Supplementary Table S4). Of the 39 gene sets for Generation R, 15 showed positive enrichment scores while 24 showed negative enrichment scores. Further, of the 414 significant gene sets for ALSPAC, 332 showed negative normalized enrichment scores indicating majority of the significant gene sets showed concordant negative association with PM_2.5_. Of the 24 that were significant and had negative enrichment scores in Generation R, 20 showed similar significant negative enrichment scores in ALSPAC (Supplementary Table S5). In agreement with the majority of the PM_2.5_-associated genes showing a negative association in Generation R, nine of the top 10 most statistically significant gene sets (Fig. 4b) showed significant negative enrichment scores and five of these top 10 gene sets also showed significant negative enrichment scores in ALSPAC as well (Fig. 5b). Among the gene sets that showed negative enrichment scores in both cohorts and were within the top 10 most significant ones by adjusted *P*-value included those originating from various immune response against external agents such as GSE13485 (genes downregulated in comparison of unstimulated PBMC after stimulation with yellow fever YF17D vaccine), GSE18791 (genes differentially expressed in dendritic cells exposed to Newcastle virus), and GSE42724 (genes up-regulated in B lymphocytes: naïve versus plasmablasts). Other than the common ones, the Generation R PM_2.5_ GSEA analysis also showed significant suppression of the microbial exposure response gene sets such as GSE14000 (unstimulated versus 4-h lipopolysaccharide exposure genes down in dendritic cells), GSE34205 (rous sarcoma virus versus flu genes up in infant PBMC) among its top 10 most significant gene sets.

## Discussion

In the present study, the main findings were that PM_2.5_ exposure was the most important environmental exposure associated with gene expression levels in children in the Generation R Study. In total, PM_2.5_ exposure was associated with expression of 86 genes, and 69 of the 86 genes showed significantly overrepresented interactions using STRING protein interaction data. Further, GO enrichment analysis showed evidence for enrichment of genes involved in innate immune response, bacterial infection, gas transport, and regulation of cell death. PM_2.5_ exposure was only associated with expression of one gene, *GNG11*, in the same direction in both studies. GSEA using both cohorts, showed significant negative enrichment of gene sets involved in microbial response including interferon alpha and gamma response. Our findings support previous studies where air pollutant affected genes enrich for immune response.

Among the 86 genes that were associated with PM_2.5_ exposure, the gene *GNG11* was also found in the independent ALSPAC Study. *GNG11* encodes a protein called G Protein Subunit Gamma 11 which is a lipid-anchored, cell membrane protein and plays a role in transmembrane signaling. In mice, the gene *GNG11* was reported to show significantly altered CpG methylation and gene expression levels in neonatal cardiomyocytes exposed to diesel exhaust pollutants compared with unexposed cells [36]. We found the expression of *GNG11* to be among the initial list of 86 genes associated with PM_2.5_ and the list of 20 genes with only a limited effect of adjustment for cell type proportions. Further functional studies in humans are needed to delineate the role of *GNG11* in the response to PM_2.5_ exposure in early life. A reason why associations of PM_2.5_ exposure with gene expression only replicated for one of the genes, *GNG11*, could be that RNA sequencing, which was used in the Generation R Study, is a more sensitive gene expression technique compared to microarray analysis, which was used in the ALSPAC Study [37]. In the Generation R Study, only 40 associated genes had a probe that could be detected in the follow-up study. Studies that have compared the two transcriptional profiling platforms have reported that a larger number of differentially expressed genes can be detected with RNA sequencing compared to microarrays and that the difference between the two platforms is largely determined by how accurately genes expressed at low levels can be quantified [38]. Since several of these 40 genes were expressed at low levels in both cohorts, and RNA sequencing is a more sensitive detection technique, this may also explain why only one of the associations of PM_2.5_ exposure with gene expression replicated.

Among the other expressed genes that were associated with PM_2.5_ exposure in Generation R, in model one at FDR < 0.25, *TSC22D1, GFI1B, CAVIN2*, and *P2RY14* is supported by previous literature as plausible candidate genes for regulation by PM_2.5_ exposure. Both *TSC22D1* and *GFI1B are genes that encodes proteins that* are transcriptional regulators and these genes have been reported to be differentially downregulated in response to PM_2.5_ and ozone in airway cells respectively [39, 40]. It is not known if epigenetic alterations affect the expression of *GFI1B* in humans, but in mice, Gfi1b can be involved in recruiting chromatin-modifying proteins and thereby epigenetically affect the differentiation of hematopoietic stem cells [41]. Interestingly, GFI1B was also among the 69 PM_2.5_ associated genes that formed a dense network using STRING protein-protein interaction database. Since the *GFI1B* gene is coding for a transcription factor expressed in hematopoietic lineage cells, this may indicate that change in expression of this gene could also have a cascading effect. The gene *CAVIN2*, enocdes a protein called Caveolae Associated Protein 2 which has been implicated in *in vitro* angiogenesis and endothelial cell proliferation, migration, and invasion. It is also reported to be downregulated in chronic intermittent hypoxia, a condition observed in obstructive sleep apnea which is reported to be associated with exposure to PM_2.5_ [42]. Lack of functional *P2RY14* gene in mice is reported to result in decreased airway eosinophilia and airway hyperresponsiveness relative to wild-type mice in mice models of asthma [43]. Of note, *TSC22D1, GFI1B*, and *CAVIN2* expression also showed a negative association with PM_2.5_ in the ALSPAC cohort but the association was not statistically significant.

The GSEA results were also consistent with PM_2.5_ association enrichment results for the 86 significant hits with 61% (24 out of 39) in Generation R and 80% (332 out of 414) in ALSPAC of significant immune gene sets showing negative enrichment scores for genes involved in microbial infection response, which is an indicator of PM_2.5_ exposure being more associated with suppression of gene expression than activation. Further 20 of the 24 significant negative gene sets in Generation R were also significant in ALSPAC. These results indicate that similar gene sets may be regulated by PM_2.5_ in both cohorts. The larger number of significant gene sets having negative enrichment scores is also consistent with previous reports of more genes showing negative association with PM_2.5_ than positive ones [40]. PM_2.5_ GSEA analysis using hallmark gene sets for both cohorts showed significant negative enrichment scores for interferon alpha and gamma response genes. Interferon alpha is type one interferon and our results are also consistent with a previous study in adults that reported a downregulation of genes involved in type one interferon response via GSEA analysis for PM_2.5_ exposure [24]. Furthermore, long-term PM_2.5_ exposure was reported to lower influenza virus resistance in mice via downregulation of pulmonary macrophage Kdm6a and histone modifications in interferon gene promoter region [44]. The GSEA analysis for both cohorts showed suppression of genes involved in response of dendritic cells in viral (GSE34205, GSE18791) and bacterial (GSE14000) infection. Several independent studies have reported that exposure to PM_2.5_ results in increased susceptibility to viral infection [45, 46]. A previous study [47] that investigated the effect of PM_2.5_ exposure versus combined PM_2.5_ and influenza viral exposure found a combined viral and PM_2.5_ exposure resulted in a greater number of virally infected cells than the only virus treatment.Combining our results with the previously mentioned study, we could speculate that PM_2.5_ exposure induces similar suppression of genes as does viral exposure.

A strength of this study is that we were able to follow-up the findings in the discovery study in an independent study with children of the same age. Further, we found that the environmental exposure distributions of the whole cohort and the sub cohort chosen for gene expression analyses weere similar possibly making our results more broadly applicable to the general population. While the previously reported candidate genes from the discovery study did not replicate in the follow-up analyses at the desired statistical significance level, several of them still showed the same direction of effect in the follow-up analyses. We thus leveraged the power of GSEA, which intends to find concordant changes in gene expression to show that across both cohorts, similar gene sets were significantly affected by PM_2.5_ exposure, despite having different sources of RNA and different methods for profiling of gene expression.

There are some limitations in our studies, notably limitations related to estimation of environmental exposures that need to be considered while interpreting our results. The current air pollution measuring system that uses LUR models is not personalized and hence there is no personal monitoring or information on duration of air pollutant exposure. In the current study, predictions from a LUR model from 2010 were applied. However, studies have documented that LUR models can be utilized successfully to estimate air pollution concentrations several years forwards or backwards in time [48]. Thus, we assume a minimal impact of this extrapolation on the level of air pollutant measurement error in our study. Further studies at different time points in childhood are necessary to determine how the transcriptomic response to PM_2.5_ varies with time and the critical age of exposure. Furthermore, due to the small sample size of the discovery cohort, we could not carry out sex-stratified analysis. Thus, studies in larger cohorts with greater statistical power are needed to follow-up our findings.

In conclusion, our study shows that PM_2.5_ exposure in early life was associated with gene expression levels, but little evidence of association was observed for the other examined exposures. Exposure to PM_2.5_ indicated a strong concordant suppression of gene sets involved in viral and bacterial response such as interferon alpha and gamma across both cohorts. Interferons play essential roles in the body’s defense against viral, bacterial, and protozoan infections, since all are important for children’s health. By affecting expression of interferon response genes, PM_2.5_ may contribute to reduced response against microbial infection as well as contribute to inflammation and oxidative stress, which are all CVD risk factors important for children later in life. Future research to strengthen our findings on PM_2.5_ exposure and expression of genes involved in viral and bacterial responses in larger cohorts are important. In addition, studies that focus on the effect of PM_2.5_ on interferon-related signaling pathways and its subsequent effect on viral load in cells would be necessary to determine if there is a causal effect of PM_2.5_ on viral load via regulation of interferon signaling. We hope our findings will lead to more investigations on how environmental exposures affect gene expression and health outcomes in children.

## Material and methods

### Cohorts and study participants

Generation R is a prospective cohort study from fetal life onwards based in the city of Rotterdam, the Netherlands [49]. The study aims to identify environmental and genetic causes of growth, development, and health. It started with the enrollment of 9778 pregnant women between April 2002 and January 2006 (61% response at baseline), who gave birth to 9901 live born children, which are enrolled in ongoing follow-up. In regular follow-up rounds (every 3–4 years), data were collected via detailed physical and behavioral observations, questionnaires, interviews as well as extensive biosampling and imaging. A subgroup of 174 children was selected for gene expression profiling using whole blood at 9 years of age, based on a homogenous European ancestry background and high availability of follow-up data. The Generation R Study protocol was approved by the Medical Ethical Committee of Erasmus MC, University Medical Center Rotterdam. Written informed consent was obtained for all participants.

ALSPAC is also a prospective cohort study of parents and children set up to provide a unique multigenerational family study and to be a resource for international researchers to explore the environmental, socioeconomic, lifestyle, physiological, metabolic, genomic, and epigenomic contributions to health and development across the life course and across generations [50–52]. Pregnant women resident in Avon, UK with expected dates of delivery between 1 April 1991 and 31 December 1992 were invited to take part in the study. The initial number of pregnant women enrolled was 14 541. Of the initial pregnancies, there was a total of 14 676 fetuses, resulting in 14 062 live births out of which 13 988 children who were alive at 1 year of age. When the oldest children were ∼7 years of age, an attempt was made to bolster the initial sample with eligible cases who had failed to join the study originally. The total sample size for analyses using any data collected after the age of 7 years is therefore 15 447 pregnancies, resulting in 15 658 fetuses. Of these, 14 901 children were alive at 1 year of age. Mothers, their partners, and children have been followed up regularly since recruitment. The study website containing details of all the data that are available through a fully searchable data dictionary and variable search tool [53]. A subset of DNA had been extracted as described previously from blood samples collected from cord blood at research clinics and lymphoblastoid cell lines were established by transforming lymphocytes from blood samples taken when the study participants were 9 years old, with Epstein–Barr Virus [54]. Ethical approval for the study was obtained from the ALSPAC Ethics and Law Committee and the Local Research Ethics Committees. Consent for biological samples has been collected in accordance with the Human Tissue Act (2004). Informed consent for the use of data collected via questionnaires and clinics was obtained from participants following the recommendations of the ALSPAC Ethics and Law Committee at the time

In total, 174 samples from Generation R and 948 samples from ALSPAC with gene expression data at around 10 years of age from whole blood and lymphoblastoid cell lines, respectively, were available. These numbers were based on availability of phenotypic data as well as budgetary constraints. Two samples were removed from the Generation R Study since they were siblings of two other children, while two samples were removed from ALSPAC since they were duplicates of each other giving a final sample size of 172 and 946 for the Generation R and ALSPAC Studies, respectively.

### Measurement of environmental exposures in Generation R and ALSPAC

Measures of air pollutant exposures including PM_2.5_, PM_10_, and NO_2_ as well as natural spaces including green space and blue space, and NDVI (as a measure of dense vegetation) in Generation R and ALSPAC were derived in the LifeCycle Project, and more details have been described elsewhere [55, 56]. In brief, exposure estimates for air pollutants were based on the (LUR modeling approach for 2010 developed in the ELAPSE Project [ [[57]57]. [81]Temporally adjusted exposure levels to each pollutant were estimated by combining the LUR spatial estimates of pollutants for their geocode with a temporal adjusting factor obtained from the routine monitoring data [58]. Estimated exposures were assigned to each study participant for their geocoded addresses through geographical information system platforms. Exposure measures corresponded to the average exposure levels in the year leading up to the child’s 9th birthday.

Vegetation index at three distance buffers (100 m, 300 m, and 500 m), and presence of major green space at <300 m from the home address were used as indicators of green space exposure. Presence of a major blue space at <300 m from the home address was used as indicators of exposure to blue space.

NDVI quantifies vegetation by measuring the difference between near-infrared (which wavelength vegetation reflects) and red light (which wavelength vegetation absorbs). NDVI was derived from the Landsat four to five Thematic Mapper (TM), Landsat seven Enhanced Thematic Mapper Plus (ETM+), and Landsat eight Operational Land Imager (OLI)/Thermal Infrared Sensor (TIRS). The imagery was selected as per the following criteria: (i) cloud cover <10%, (ii) Standard Terrain Correction (level one T), and (iii) greenest period of the year, for best image contrast. Distance, in meters, to the nearest green or blue major space, >5000 m^2^, and size of the respective natural spaces were extracted from the Europe-wide “‘Urban Atlas” [59].

### Measurement of maternal education and area SES in Generation R

Based on a questionnaire used at enrolment, the highest education achieved by each mother was established as previously described [60]. This was categorized into three levels for analysis purpose including low education, secondary education, and high education.

Area SES determination has been previously described [61]. Briefly, the neighborhood zip code was obtained by parent-reported questionnaires at birth and at child age of 9 years. Information on neighborhood SES was obtained from the Netherlands Institute of Social Research (SCP). Neighborhood SES scores were computed by SCP in 2002, 2006, 2010, 2014, 2016, and 2017 with principal component analysis based on the mean resident income, percentage of low resident incomes, percentage of low educated residents, and percentage of unemployed residents in a neighborhood. The nearest data available on neighborhood SES scores were matched to the neighborhood zip code at birth (2006), at child aged 6 years (2010), and at child aged 13 years (2017). Participants were categorized in three groups according to their neighborhood SES scores: low neighborhood SES (1st tertile), middle neighborhood SES (2nd tertile), and high neighborhood SES (3rd tertile).

### Measurement of maternal education and area SES in ALSPAC

Maternal education and area SES was determined as described previously [51]. Information on the highest qualification of the mother was obtained at recruitment, during pregnancy. Maternal level of education was based on the highest ongoing or completed education and classified as low, medium, and high according to the International Standard Classification of Education 97/2011 (ISCED-97/2011). High education corresponded to short cycle tertiary, Bachelor, Masters, Doctoral or equivalent (ISCED2011: 5—8, ISCED-97: five5—6); medium education corresponded to upper secondary, or post-secondary non-tertiary (ISCED-2011: 3–-4, ISCED-97: 3—4); and low education corresponded to no education; early childhood; preprimary; primary; lower secondary or second stage of basic education (ISCED-2011: 0–2, ISCED-97: 0—2).

Area SES in ALSPAC was measured as area deprivation based on data from the Ministry of Housing, Communities & Local Government (UK Government) from 2015 and linked to geocoded addresses at birth. It combined weighted information from seven domains to produce an overall relative measure of deprivation. The domains and their weights were the following: income deprivation (22.5%), employment deprivation (22.5%), education, skills, and training deprivation (13.5%), health deprivation and disability (13.5%), crime (9.3%), barriers to housing and services (9.3%), and living environment deprivation (9.3%). It was categorized in quintiles, where the first corresponds to the least deprived and the last to the most deprived.

### RNA isolation and processing of RNA sequencing data for Generation R

The RNA isolation was carried out at the Erasmus MC, University Medical Center Rotterdam by trained staff. Briefly, whole blood was collected in PAXgene tubes and stored at −20°C before RNA isolation. The Maxwell^®^ RSC miRNA Tissue kits and Maxwell^®^ RSC instrument (artnr. AS1460 and AS4500, respectively; Promega, Madison, WI, USA) were used for extraction of total RNA, including microRNA. Total RNA was frozen immediately after isolation and stored at −80°C. Total RNA from whole blood was depleted of globin transcripts using the Ambion GLOBINclear kit (Thermofisher Scientific, CA, USA). RNA concentration was measured with the NanoDrop (ND-8000, Thermo Scientific™) and RNA integrity was measured with the DNA 5 K/RNA Labchip assay (artnr. 760 435) and RNA Assay Reagent kit (artnr. CL2960010) on the LabChip GX Touch (Perkin Elmer Sequencing library was prepared using the Illumina TruSeq RNA Library Preparation Kit (Illumina, CA, USA). Paired-end sequencing of 2x150-bp reads was performed using the Illumina HiSeq 4000 platform, with a total of about 4 Gb raw-sequencing data per sample.

Raw sequencing reads were processed by first aligning to the UCSC hg19 reference genome using STAR (v2.5.0c) two-pass methodology [62], taking splice junctions into account. Aligned reads in binary alignment map (BAM) files were then postprocessed by removing hemoglobin-aligned reads using samtools, v1.10 [63], sorted by genomic coordinate, and marked duplicates using Picard (v2.26.2) [64] followed by indel realignment and base quality score recalibration using Genome Analysis ToolKit (v3.5) [65]. Quality control metrics were extracted at various steps using Picard and GATK. Read counting was then performed on the processed BAM files using featureCounts [66] based on the GENCODE version 38 gene annotations, lifted over to hg19. Reads were counted at exon level to obtain counts by gene for all samples.

### RNA isolation, microarray hybridization, and data processing for ALSPAC

The generation of the gene expression data in ALSPAC has been described previously [54]. Briefly, lymphoblastoid cell lines (LCLs) from unrelated individuals were grown in identical conditions, and RNA was extracted using RNeasy extraction kit (Qiagen, Hilden, Germany) and amplified using Illumina TotalPrep-96 RNA amplification kit (Illumina, CA, USA) as previously described. Expression profiling of the samples, with two technical replicates, were performed using the Illumina HT-12 V3 BeadChips (Illumina, CA, USA) including 48 804 probes mapping to hg18 version of the human genome. A total of 200 ng of total RNA was processed according to supplier’s instructions. Raw data were imported to Illumina Beadstudio software to transform bead-level data to probe-level intensities. Probes with less than three beads present were excluded. These data were subsequently exported for bioinformatic analyses. Log_2_-transformed expression probe-level intensities were quantile normalized first within replicates of each individual followed by quantile normalization across all individuals using the preprocessCore::normalize.quantiles() function. Initial analysis was initially restricted to 24 140 probes uniquely tagging to genes annotated in the Ensembl database.

### Association of environmental exposures with gene expression

We used the Generation R RNA sequencing gene expression data as the discovery dataset and the ALSPAC microarray gene expression data for follow-up analysis for two reasons. Firstly, RNA sequencing is a newer technology for measuring gene expression relative to microarray and is reported to have a higher sensitivity of detecting gene expression and consequently detection of differentially expressed including lowly expressed genes compared to microarrays [67, 68]. Secondly, it has previously been reported that viral transformation of the B cells into indefinitely proliferating lymphoblastoid cell lines *in vitro* could interrupt host genome gene expression activity, which would not be the case for untransformed whole blood derived transcriptome data [69].

For both datasets, we restricted the analysis to genes and probes mapping to autosomes. For the Generation R RNA sequencing data, we filtered the raw count data of the 172 samples to keep genes with at least two counts mapping to them in at least 50% of the samples, giving a total of 18 787 genes. The trimmed method of means (TMM) normalization implemented in the edgeR [70] R package v3.40.2 was used to account for differences in sequencing library size and composition. The negative binomial test implemented in edgeR was used to test for association of exposures with gene expression adjusting for covariates. For the ALSPAC microarray gene expression data, we used the batch corrected data that was filtered to keep probes with average log expression greater than 7.5, resulting in a total of 14 054 probes from an initial count of 24 140 probes. The quantile normalized microarray data were subsequently analyzed using the limma R package v3.54.2 [71], where the limma::lmFit() function was for model fitting and the limma::empirical Bayes test for univariate association analysis of exposure and gene expression levels.

In both cohorts, to control for confounding, we adjusted for sex and age. We used RNA sequencing flow cell identification as the batch variable for the Generation R cohort. We additionally adjusted for maternal education and area SES to account for any residual confounding. Maternal education has been previously reported to affect gene expression during pregnancy and *in utero* [72] and area SES affects gene expression in PBMCs [73]. For the maternal education variable, due to a small sample size of the Generation R cohort, the individuals were grouped into below post-secondary education versus post-secondary education or above. No multiple imputation was done due to the exploratory nature of the analysis and the relatively low rate of missingness to no missingness in the discovery cohort. All participants in this study were of European ancestry and hence no adjustment for ethnicity was done. We also adjusted for cell-type composition differences across samples in the Generation R Study. In the Generation R Study, RNA was isolated from whole blood which is composed of multiple different cell types, and cell types were estimated using the Houseman method with the Reinius reference on existing DNA methylation data, as previously described [74]. There was no adjustment for cell-type differences in the ALSPAC Study since the lymphoblastoid cells originate from peripheral blood lymphocytes only. All quantitative exposure variables were mean centered before fitting the models. Below is the summary of the models used in the association of gene expression with environmental exposures:

Model 1: Gene expression ∼ Exposure + Age + Sex + Batch + Maternal education + Area statuses.

Model 2: Gene expression ∼ Exposure + Age + Sex + Batch + Maternal education + Area socioeconomic status + Cell-type composition.

Due to the exploratory nature of the study and the relatively small sample size in the discovery analysis, we reported genes that were associated with the exposures using a Benjamini Hochberg FDR-corrected *P*-value < 0.25 in Models 1 and 2 specified above. The cell-type composition was only available for 121 samples in Generation R, reducing the power for discovery. To detect if the significant genes in Model 1 was not confounded by cell-type differences across samples, we considered genes from Model 1 at FDR <0.25 which showed a <10% percentage change in regression coefficient in Model 2 compared to Model 1 and used nominal *P*-value at <.05 in Model 2. For the follow-up analysis using the ALSPAC Study, we used the complete dataset for determining the unadjusted *P*-value using the empirical Bayes approach of limma. We subset to the genes of interest from Generation R before multiple testing correction in ALSPAC and used a more stringent cutoff of 0.05 for the FDR-corrected *P*-value to follow-up on the candidate genes of interest from Generation R.

### STRING DB protein–protein interaction analysis

Gene expression measurements are often done as a proxy for protein levels. Thus, we leveraged the STRING protein-protein interaction network database data to find if genes associated with select environmental exposures are known to interact at the protein level [75]. These interactions are based on genomic context predictions, high-throughput lab experiments, co-expression data, automated text mining, and previous knowledge databases like Biocarta, BioCyc, GO, KEGG, & Reactome. We used the STRING database online tool to determine if the protein products of the environmental exposure-associated genes are predicted to interact at the protein level. STRING generates an expected number of interactions using a user-supplied background gene set and uses a hypergeometric test to determine if there are greater number of interactions than expected by random. On the STRING webpage [76], the gene symbols for the genes showing suggestive association with an exposure of interest were entered and resulting visualized network was downloaded.

### Gene ontology and GSEA

For the Generation R cohort, associated genes with an adjusted *P*-value <.25 were used for GO enrichment analysis. The GO enrichment terms were derived from the Molecular Signatures Database (MSigDB) [77]. The clusterProfiler [78] R package version 4.6.2 was used for enrichment analysis using the “enrichGO” function and the threshold for significant terms was set at 0.01 after FDR correction. The MSigDB is one of the most widely used and comprehensive databases of gene sets for performing GSEA. The latest version of MSigDB consists of multiple collections of gene sets of which we used the hallmark gene sets and immunological condition gene sets. The enrichGO function uses a Fisher’s exact test to determine if a gene set from the GO database is overrepresented in a differential expression analysis generated significant gene list compared to the number of genes annotated to each GO term which forms part of the background. We carried out GO enrichment analysis only if the minimum number of associated genes (FDR < 0.25) was 10 so that the analysis would be sufficiently powered to detect significantly enriched terms.

Using the ranked list of regression coefficients for all the genes from the differential analysis results for select exposures, GSEA was carried out in both cohorts using the clusterProfiler R package with the MSigDB hallmark and immunologic signature database terms [79]. GSEA is a rank-based procedure that tests to see if genes belonging to a gene set fall mostly near the top of the list indicating most activated or towards the bottom indicating most suppressed. It uses a priori predefined gene sets applied on a ranked list of coefficients, which in this case are the regression coefficients of all genes from the differential expression analysis of any exposure using edgeR or limma. It calculates an enrichment score that represents the amount to which the genes in the set are over-represented at either the top or bottom of the list. Subsequently, the scores are normalized by the gene set size. It then uses a phenotype-based permutation test to produce a null distribution for the enrichment score and then determines the *P*-value by comparing the enrichment score to the null distribution of scores. A significant negative score indicates that most genes in that gene set are negatively associated with the exposure while a positive score indicates most of the genes are positively associated with the exposure. The hallmark gene sets contain 50 gene sets that represent well-defined biological states with coherent expression patterns and due to the overall small gene set size is considered a good first pass to get biological insights using GSEA. The immunologic collection consists of 4872 gene sets that represent cell states and perturbations within the immune system with data from various *in vitro* or *in vivo* experimental setups and thus are ideal for generating plausible hypotheses in a transcriptomic study with no upstream perturbation information. We used the Model 2 derived coefficients for Generation R such that the differences in cell-type composition are accounted for and the ranking of genes for the PM_2.5_ coefficient in the regression models is not influenced by it. For ALSPAC, we used the coefficients from the only model we generated for it. The threshold for multiple testing corrections for the terms were set at 0.01.

## Supplementary Material

dvae025_Supp

## Data Availability

All queries regarding availability of the Generation R data should be addressed to Dr Janine Felix while the ALSPAC Study management committee can be contacted for accessing the data from the ALSPAC cohort.
