# Supplementary material for: Air pollution exposure is associated with gene expression in children
Source: Environ Epigenet. 2024 Dec 21;10(1):dvae025. doi: 10.1093/eep/dvae025 (PMC11668970; doi:10.1093/eep/dvae025)
Supplement: dvae025_Supp [file dvae025_supp.zip › suppl_data/Supplementary Table 2.pdf]

Supplementary Table 2: $PM_{2.5}$ associated 86 genes from model 1 of Generation R cohort

logFC	logCPM	LR	PValue	p.adjusted	Gene	Symbol
-0.55	1.79	20.68	5.43E-06	0.051	ENSG00000130300	PLVAP
-0.44	6.32	21.15	4.25E-06	0.051	ENSG00000156265	MAP3K7CL
-0.76	1.56	19.13	1.22E-05	0.054	ENSG00000235169	SMIM1
-0.43	3.97	18.78	1.47E-05	0.054	ENSG00000137198	GMPR
-0.29	3.1	18.46	1.73E-05	0.054	ENSG00000008441	NFIX
-0.4	3.89	19	1.30E-05	0.054	ENSG00000088826	SMOX
-0.34	7.93	18.09	2.11E-05	0.057	ENSG00000100225	FBXO7
-0.35	3.07	17.78	2.47E-05	0.058	ENSG00000082781	ITGB5
-0.52	2.44	17.24	3.29E-05	0.066	ENSG00000198892	SHISA4
-0.85	6.03	17.12	3.51E-05	0.066	ENSG00000266037	NULL
-0.24	5.11	16.56	4.72E-05	0.074	ENSG00000111644	ACRBP
-0.41	9.08	16.72	4.34E-05	0.074	ENSG00000013306	SLC25A39
-0.29	3.23	16.05	6.19E-05	0.083	ENSG00000170271	FAXDC2
-0.31	2.65	16.18	5.77E-05	0.083	ENSG00000124635	H2BC11
-0.36	1.75	15.9	6.68E-05	0.084	ENSG00000279841	AC092135.3
-0.36	3.89	15.78	7.13E-05	0.084	ENSG00000162722	TRIM58
-0.51	4.73	15.11	0.00010146	0.085	ENSG00000143416	SELENBP1
-0.87	-0.4	14.43	0.00014556	0.085	ENSG00000180999	C1orf105
-0.24	7.97	14.58	0.00013432	0.085	ENSG00000159346	ADIPOR1
-0.25	3.68	14.45	0.00014383	0.085	ENSG00000168785	TSPAN5
-0.35	5.84	14.88	0.00011429	0.085	ENSG00000127920	GNG11
-0.39	6.73	14.55	0.00013651	0.085	ENSG00000158856	DMTN
-0.28	6.73	14.53	0.00013775	0.085	ENSG00000107262	BAG1
-0.72	5.47	14.73	0.00012382	0.085	ENSG00000213934	HBG1
-0.53	3	14.95	0.00011064	0.085	ENSG00000167768	KRT1
-0.45	4.97	14.7	0.0001261	0.085	ENSG00000169385	RNASE2
-0.48	0.27	14.84	0.00011713	0.085	ENSG00000285774	SAMD4A-AS1
-0.26	4.24	15.59	7.87E-05	0.085	ENSG00000248334	WHAMMP2
-0.48	3.04	15.37	8.85E-05	0.085	ENSG00000166947	EPB42
-0.93	-1.08	15.07	0.00010366	0.085	ENSG00000140986	RPL3L
-0.49	5.21	15.36	8.88E-05	0.085	ENSG00000004939	SLC4A1
-0.41	1.94	14.92	0.00011197	0.085	ENSG00000267279	AC090409.1
-0.23	4.97	14.16	0.00016822	0.096	ENSG00000124098	FAM210B
-0.82	0.34	13.83	0.00020018	0.107	ENSG00000196565	HBG2
-0.43	0.43	13.86	0.00019726	0.107	ENSG00000172927	MYEOV
-0.3	7.02	13.71	0.00021376	0.112	ENSG00000060138	YBX3

-0.41	0.52	13.38	0.00025476	0.122	ENSG00000237276	ANO7L1
-0.29	1.94	13.22	0.00027757	0.122	ENSG00000153162	BMP6
-0.13	7.79	13.26	0.00027064	0.122	ENSG00000146278	PNRC1
-0.14	7.27	13.27	0.00026926	0.122	ENSG00000136819	C9orf78
-0.59	2.96	13.16	0.00028665	0.122	ENSG00000225698	IGHV3-72
-0.44	4.37	13.32	0.00026275	0.122	ENSG00000169877	AHSP
-0.35	10.68	13.28	0.00026803	0.122	ENSG00000105701	FKBP8
-0.65	0.27	13.16	0.00028566	0.122	ENSG00000253818	IGLV1-41
-0.31	5.66	13.07	0.00029981	0.125	ENSG00000168497	CAVIN2
-0.26	4.03	12.79	0.00034847	0.131	ENSG00000174944	P2RY14
-0.41	2.23	12.84	0.00033892	0.131	ENSG00000223855	PDGFA-DT
-0.31	4.07	12.89	0.00032952	0.131	ENSG00000165702	GFI1B
-0.26	4.29	12.82	0.00034262	0.131	ENSG00000108960	MMD
-0.53	0.04	12.86	0.00033481	0.131	ENSG00000266401	LOC105371967
-0.42	1.19	12.71	0.00036417	0.134	ENSG00000238243	OR2W3
-0.32	8.13	12.62	0.00038136	0.138	ENSG00000136732	GYPC
-0.3	3.51	12.54	0.00039799	0.141	ENSG00000204020	LIPN
-0.3	5.14	12.49	0.0004083	0.142	ENSG00000082146	STRADB
0.21	1.92	12.46	0.00041564	0.142	ENSG00000211749	TRBV23-1
-0.32	3.45	12.3	0.00045375	0.142	ENSG00000187699	C2orf88
-0.33	4.8	12.32	0.00044847	0.142	ENSG00000145335	SNCA
-0.17	4.59	12.36	0.00043778	0.142	ENSG00000102804	TSC22D1
0.75	-1.9	12.32	0.00044761	0.142	ENSG00000259709	AC023906.4
-0.51	4.14	12.42	0.00042379	0.142	ENSG00000086506	HBQ1
0.23	1.99	12.21	0.00047593	0.147	ENSG00000170469	SPATA24
-0.25	2.73	12.14	0.00049346	0.147	ENSG00000162367	TAL1
-0.17	6.16	12.15	0.00049192	0.147	ENSG00000198876	DCAF12
-0.32	3.8	11.93	0.00055292	0.162	ENSG00000184792	OSBP2
0.5	-0.59	11.86	0.00057495	0.166	ENSG00000123892	RAB38
-0.41	4.93	11.69	0.00062776	0.169	ENSG00000162366	PDZK1IP1
-0.34	3.51	11.69	0.00062742	0.169	ENSG00000079308	TNS1
-0.21	3.63	11.68	0.00063086	0.169	ENSG00000229754	CXCR2P1
-0.42	4.05	11.74	0.00061133	0.169	ENSG00000108309	RUNDC3A
-0.15	6.91	11.77	0.00060238	0.169	ENSG00000100325	ASCC2
-0.37	2.46	11.48	0.00070351	0.186	ENSG00000151023	ENKUR
-0.23	5.57	11.32	0.00076499	0.2	ENSG00000183625	CCR3
-0.18	6.4	11.26	0.0007914	0.204	ENSG00000175857	GAPT
-0.17	4.64	11.23	0.00080453	0.204	ENSG00000182512	GLRX5
-0.5	0.43	11.06	0.00088398	0.221	ENSG00000211645	IGLV1-50

-0.32	6.37	11	0.00091291	0.226	ENSG00000154146	NRGN
-0.25	2.87	10.97	0.00092465	0.226	ENSG00000276141	WHAMMP3
-0.34	1.5	10.91	0.00095494	0.227	ENSG00000227165	WDR11-DT
-0.25	3.13	10.92	0.00095107	0.227	ENSG00000185340	GAS2L1
-0.37	-0.15	10.87	0.00097797	0.23	ENSG00000267541	MTCO2P2
0.44	-1.63	10.83	0.00099948	0.232	ENSG00000264756	NULL
-0.33	1.36	10.77	0.001033	0.236	ENSG00000172889	EGFL7
-0.41	0.9	10.75	0.00104308	0.236	ENSG00000134548	SPX
-0.28	3.95	10.67	0.00108977	0.244	ENSG00000088726	TMEM40
0.3	-0.09	10.59	0.001135	0.25	ENSG00000160678	S100A1
-0.58	1.96	10.58	0.00114365	0.25	ENSG00000169397	RNASE3
