# Supplementary material for: Air pollution exposure is associated with gene expression in children
Source: Environ Epigenet. 2024 Dec 21;10(1):dvae025. doi: 10.1093/eep/dvae025 (PMC11668970; doi:10.1093/eep/dvae025)
Supplement: dvae025_Supp [file dvae025_supp.zip › suppl_data/Supplementary Table 3.pdf]

Supplementary Table 3: $PM_{2.5}$ association results for 86 genes in model 2 that were also significant in model 1

logFC	logCPM	LR	PValue	p.adjusted	Gene	Symbol
-0.37	4.25	15.45	8.47E-05	0.796	ENSG00000248334	NULL
-1.24	-1.27	12.99	0.000312889	0.986	ENSG00000140986	RPL3L
-0.83	5.59	10.53	0.001177653	0.986	ENSG00000213934	HBG1
-0.45	6.33	9.86	0.001688644	0.986	ENSG00000156265	MAP3K7CL
-0.5	4.93	9.65	0.001889862	0.986	ENSG00000169385	RNASE2
-0.37	5.84	9.44	0.002123808	0.986	ENSG00000127920	GNG11
-1.1	-0.41	9.41	0.002161792	0.986	ENSG00000180999	C1orf105
-0.16	7.27	8.99	0.002720616	0.986	ENSG00000136819	C9orf78
-0.21	6.39	8.35	0.003849993	0.986	ENSG00000175857	GAPT
-0.36	3.88	8.21	0.004167312	0.986	ENSG00000088826	SMOX
-0.3	2.89	7.92	0.00488598	0.986	ENSG00000276141	NULL
-0.24	5.55	7.91	0.004903565	0.986	ENSG00000183625	CCR3
0.25	2.04	7.64	0.00572211	0.986	ENSG00000170469	SPATA24
-0.33	5.66	7.42	0.006451723	0.986	ENSG00000168497	CAVIN2
-0.26	3.98	7.35	0.00671222	0.986	ENSG00000174944	P2RY14
-0.23	4.96	6.9	0.008609244	0.986	ENSG00000124098	FAM210B
-0.18	4.59	6.85	0.008882969	0.986	ENSG00000102804	TSC22D1
-0.8	0.49	6.73	0.009475071	0.986	ENSG00000196565	HBG2
-0.64	1.9	6.72	0.00951381	0.986	ENSG00000169397	RNASE3
-0.26	4.29	6.69	0.009701136	0.986	ENSG00000108960	MMD
-0.48	2.45	6.61	0.010161593	0.986	ENSG00000198892	SHISA4
0.77	-1.79	6.53	0.010598738	0.986	ENSG00000259709	NULL
-0.23	3.65	6.23	0.012553171	0.986	ENSG00000229754	NULL
-0.3	7.93	6.18	0.01294012	0.986	ENSG00000100225	FBXO7
-0.32	3.46	5.95	0.014746796	0.986	ENSG00000187699	C2orf88
0.45	-0.58	5.81	0.015974975	0.986	ENSG00000123892	RAB38
-0.24	3.18	5.75	0.016524386	0.986	ENSG00000170271	FAXDC2
-0.19	7.96	5.74	0.016555194	0.986	ENSG00000159346	ADIPOR1
-0.28	1.92	5.73	0.016645028	0.986	ENSG00000153162	BMP6
-0.36	1.95	5.64	0.017572064	0.986	ENSG00000267279	NULL
-0.28	4.09	5.57	0.018302618	0.986	ENSG00000165702	GFI1B
-0.54	1.48	5.51	0.018873284	0.986	ENSG00000235169	SMIM1
-0.3	3.97	5.49	0.019155404	0.986	ENSG00000088726	TMEM40
-0.31	1.79	5.31	0.021224182	0.986	ENSG00000279841	NULL
-0.26	2.65	5.2	0.022526436	0.986	ENSG00000124635	H2BC11
-0.39	4	5.15	0.023238692	0.986	ENSG00000108309	RUNDC3A
-0.22	6.7	4.83	0.028033747	0.986	ENSG00000107262	BAG1

-0.19	5.12	4.78	0.02884782	0.986	ENSG00000111644	ACRBP
-0.36	2.44	4.75	0.029260927	0.986	ENSG00000151023	ENKUR
-0.46	2.96	4.72	0.02981837	0.986	ENSG00000167768	KRT1
-0.36	0.89	4.63	0.031326908	0.986	ENSG00000134548	SPX
-0.33	0.56	4.55	0.032962356	0.986	ENSG00000237276	NULL
-0.54	0.42	4.52	0.033585057	0.986	ENSG00000253818	IGLV1-41
0.38	-1.55	4.43	0.035268776	0.986	ENSG00000264756	NULL
-0.3	3.95	4.42	0.035519513	0.986	ENSG00000137198	GMPR
-0.36	1.73	4.24	0.039547283	0.986	ENSG00000130300	PLVAP
-0.19	3.63	4.21	0.040160433	0.986	ENSG00000168785	TSPAN5
-0.24	5.13	4.19	0.04063061	0.986	ENSG00000082146	STRADB
-0.37	4.37	3.98	0.046059959	0.986	ENSG00000169877	AHSP
-0.21	3.47	3.97	0.046262054	0.986	ENSG00000204020	LIPN
-0.26	8.1	3.95	0.046777951	0.986	ENSG00000136732	GYPC
-0.34	-0.14	3.92	0.047789705	0.986	ENSG00000267541	MTCO2P2
-0.21	7	3.62	0.057221248	0.986	ENSG00000060138	YBX3
-0.24	3.07	3.6	0.057639195	0.986	ENSG00000082781	ITGB5
-0.27	9.06	3.55	0.059431402	0.986	ENSG00000013306	SLC25A39
-0.27	6.69	3.51	0.060823953	0.986	ENSG00000158856	DMTN
-0.32	0.42	3.46	0.062771048	0.986	ENSG00000172927	MYEOV
-0.27	1.5	3.4	0.065008059	0.986	ENSG00000227165	WDR11-DT
-0.29	2.19	3.4	0.065127517	0.986	ENSG00000223855	PDGFA-DT
-0.41	0.07	3.29	0.069650517	0.986	ENSG00000266401	LOC105371967
-0.22	10.65	3.29	0.069692134	0.986	ENSG00000105701	FKBP8
-0.23	6.33	3.14	0.076411004	0.986	ENSG00000154146	NRGN
-0.17	3.09	3.05	0.08063415	0.986	ENSG00000008441	NFIX
-0.23	4.78	3.02	0.082113224	0.986	ENSG00000145335	SNCA
-0.31	3	2.99	0.083868594	0.986	ENSG00000166947	EPB42
-0.17	2.69	2.95	0.085750421	0.986	ENSG00000162367	TAL1
-0.3	0.21	2.93	0.087058075	0.986	ENSG00000285774	NULL
-0.22	3.77	2.84	0.09193071	0.986	ENSG00000184792	OSBP2
-0.21	3.86	2.82	0.093115527	0.986	ENSG00000162722	TRIM58
-0.17	3.08	2.72	0.09929805	0.986	ENSG00000185340	GAS2L1
-0.27	1.17	2.48	0.115001903	0.986	ENSG00000238243	OR2W3
-0.27	4.9	2.46	0.116763467	0.986	ENSG00000162366	PDZK1IP1
0.21	0	2.44	0.118072446	0.986	ENSG00000160678	S100A1
-0.3	4.7	2.37	0.123981162	0.986	ENSG00000143416	SELENBP1
-0.3	4.06	2.35	0.125632411	0.986	ENSG00000086506	HBQ1
-0.27	5.18	2.28	0.131327649	0.986	ENSG00000004939	SLC4A1
-0.09	6.11	2.24	0.134248954	0.986	ENSG00000198876	DCAF12
0.1	1.96	1.9	0.167688114	0.986	ENSG00000211749	TRBV23-1

-0.19	1.37	1.76	0.184632006	0.986	ENSG00000172889	EGFL7
-0.18	3.52	1.58	0.20838427	0.986	ENSG00000079308	TNS1
-0.05	7.76	1.33	0.24813948	0.986	ENSG00000146278	PNRC1
-0.08	4.61	1.33	0.248262234	0.986	ENSG00000182512	GLRX5
-0.07	6.9	1.1	0.294253304	0.986	ENSG00000100325	ASCC2
-0.15	0.52	0.47	0.492082195	0.986	ENSG00000211645	IGLV1-50
-0.09	2.96	0.18	0.675537781	0.987	ENSG00000225698	IGHV3-72
-0.09	5.92	0.09	0.761680386	0.991	ENSG00000266037	NULL
