# Supplementary material for: Air pollution exposure is associated with gene expression in children
Source: Environ Epigenet. 2024 Dec 21;10(1):dvae025. doi: 10.1093/eep/dvae025 (PMC11668970; doi:10.1093/eep/dvae025)
Supplement: dvae025_Supp [file dvae025_supp.zip › suppl_data/Supplementary Table 4 both together ALSAC 50.pdf]

Supplementary Table 4: Generation R Immunologic gene set GSEA signifivant gene sets

Gene.Set	Enrichment Score	NES	pvalue	p.adjust
GSE9006_TYPE_1_VS_TYPE_2_DIABETES_PBMC_AT_DX_UP	0.51	2.2	1.01E-10	4.92E-07
GSE13485_DAY1_VS_DAY7_YF17D_VACCINE_PBMC_DN	-0.53	-2.02	3.52E-09	8.57E-06
GSE13485_DAY3_VS_DAY7_YF17D_VACCINE_PBMC_DN	-0.52	-1.96	5.19E-08	8.43E-05
GSE18791_UNSTIM_VS_NEWCATSLE_VIRUS_DC_18H_DN	-0.54	-2	8.14E-08	9.92E-05
GSE21360_NAIVE_VS_QUATERNARY_MEMORY_CD8_TCELL_UP	-0.47	-1.82	2.12E-06	0.002
GSE34205_RSV_VS_FLU_INF_INFANT_PBMC_UP	-0.51	-1.89	7.14E-06	0.005
GSE18791_CTRL_VS_NEWCASTLE_VIRUS_DC_10H_DN	-0.47	-1.8	6.69E-06	0.005
GSE14000_UNSTIM_VS_4H_LPS_DC_DN	-0.47	-1.81	9.36E-06	0.005
GSE42724_NAIVE_BCELL_VS_PLASMABLAST_UP	-0.46	-1.77	9.21E-06	0.005
GSE19888_ADENOSINE_A3R_INH_VS_ACT_WITH_INHIBITOR_PRETREATMENT_IN_MAST_CELL_UP	-0.49	-1.83	1.79E-05	0.007
GSE14000_UNSTIM_VS_4H_LPS_DC_TRANSLATED_RNA_DN	-0.47	-1.78	1.72E-05	0.007
GSE21360_NAIVE_VS_QUATERNARY_MEMORY_CD8_TCELL_DN	-0.46	-1.78	1.59E-05	0.007
GSE37533_PPARG1_FOXP3_VS_FOXP3_TRANSDUCED_CD4_TCELL_DN	-0.46	-1.77	2.17E-05	0.008
GSE2770_TGFB_AND_IL4_ACT_VS_ACT_CD4_TCELL_2H_DN	-0.46	-1.75	5.24E-05	0.0169
GSE9006_HEALTHY_VS_TYPE_2_DIABETES_PBMC_AT_DX_UP	0.41	1.73	5.54E-05	0.0169
GSE11057_CD4_CENT_MEM_VS_PBMC_UP	0.41	1.73	5.13E-05	0.0169
GSE21678_WT_VS_FOXO1_FOXO3_KO_TREG_DN	0.41	1.72	9.33E-05	0.0227
GSE42021_TREG_VS_TCONV_PLN_UP	-0.45	-1.71	8.64E-05	0.0227
GSE18791_UNSTIM_VS_NEWCATSLE_VIRUS_DC_10H_DN	-0.45	-1.69	9.33E-05	0.0227
GSE18791_CTRL_VS_NEWCASTLE_VIRUS_DC_6H_DN	-0.44	-1.69	7.96E-05	0.0227
GSE360_L_DONOVANI_VS_M_TUBERCULOSIS_DC_UP	0.41	1.71	0.00012456	0.0256
GSE26495_NAIVE_VS_PD1LOW_CD8_TCELL_UP	0.4	1.69	0.00012641	0.0256
GSE360_L_DONOVANI_VS_T_GONDII_MAC_DN	0.4	1.68	0.00012267	0.0256
GSE40685_TREG_VS_FOXP3_KO_TREG_PRECURSOR_UP	-0.43	-1.66	0.00013139	0.0256
GSE6269_HEALTHY_VS_E_COLI_INF_PBMC_UP	0.4	1.65	0.00011892	0.0256
GSE21546_WT_VS_SAP1A_KO_DP_THYMOCYTES_UP	-0.44	-1.66	0.00013753	0.0258
GSE34205_HEALTHY_VS_RSV_INF_INFANT_PBMC_DN	-0.44	-1.69	0.00016006	0.0273
GSE19888_ADENOSINE_A3R_ACT_VS_TCELL_MEMBRANES_ACT_AND_A3R_INH_PRETREAT_IN_MAST_CELL_DN	0.4	1.67	0.00016244	0.0273
GSE10325_CD4_TCELL_VS_LUPUS_CD4_TCELL_DN	-0.43	-1.65	0.00015825	0.0273
GSE9006_TYPE_1_VS_TYPE_2_DIABETES_PBMC_AT_DX_DN	-0.43	-1.66	0.00024704	0.0348
GSE14415_INDUCED_TREG_VS_FOXP3_KO_INDUCED_TREG_IL2_CULTURE_UP	-0.44	-1.66	0.0002323	0.0348
GSE22589_HEALTHY_VS_SIV_INFECTED_DC_UP	0.39	1.65	0.00022547	0.0348
GSE11057_PBMC_VS_MEM_CD4_TCELL_DN	0.39	1.65	0.0002441	0.0348
GSE13485_DAY1_VS_DAY3_YF17D_VACCINE_PBMC_DN	-0.42	-1.62	0.0002502	0.0348
GSE10325_BCELL_VS_MYELOID_UP	0.38	1.62	0.00023369	0.0348

GSE9601_UNTREATED_VS_PI3K_INHIBITOR_TREATED_HCMV_INF_MONOCYTE_UP	0.4	1.66	0.00029295	0.0396
GSE36078_UNTREATED_VS_AD5_INF_MOUSE_LUNG_DC_UP	0.42	1.69	0.00032935	0.0422
GSE45837_WT_VS_GFI1_KO_PDC_DN	0.39	1.65	0.00032182	0.0422
GSE18791_CTRL_VS_NEWCASTLE_VIRUS_DC_8H_DN	-0.44	-1.65	0.00034096	0.0426

Supplementary Table 4 continued: Top 50 most ALSPAC Immunologic gene set GSEA significant gene sets

Gene.Set	EnrichmentScore	NES	pvalue	p.adjust
GSE13485_DAY1_VS_DAY7_YF17D_VACCINE_PBMC_DN	-0.56	-2.54	7.74E-16	0
GSE18791_UNSTIM_VS_NEWCATSLE_VIRUS_DC_6H_DN	-0.53	-2.4	1.70E-12	0
GSE10325_CD4_TCELL_VS_LUPUS_CD4_TCELL_DN	-0.52	-2.38	3.37E-12	0
GSE360_L_DONOVANI_VS_M_TUBERCULOSIS_DC_DN	-0.51	-2.3	1.23E-10	0
GSE13485_PRE_VS_POST_YF17D_VACCINATION_PBMC_DN	-0.49	-2.25	1.25E-10	0
GSE13485_CTRL_VS_DAY7_YF17D_VACCINE_PBMC_DN	-0.49	-2.25	1.40E-10	0
GSE37533_PPARG1_FOXP3_VS_FOXP3_TRANSDUCED_CD4_TCELL_DN	-0.49	-2.23	1.23E-10	0
GSE18791_UNSTIM_VS_NEWCATSLE_VIRUS_DC_10H_DN	-0.5	-2.27	2.11E-10	0
GSE1432_CTRL_VS_IFNG_24H_MICROGLIA_DN	-0.5	-2.26	1.96E-10	0
GSE42724_NAIVE_BCELL_VS_PLASMABLAST_UP	-0.5	-2.26	2.40E-10	0
GSE13484_UNSTIM_VS_YF17D_VACCINE_STIM_PBMC_DN	-0.49	-2.22	2.85E-10	0
GSE42021_CD24HI_VS_CD24INT_TREG_THYMUS_DN	-0.48	-2.21	2.74E-10	0
GSE34006_A2AR_KO_VS_A2AR_AGONIST_TREATED_TREG_UP	-0.48	-2.19	1.13E-09	0
GSE14000_UNSTIM_VS_4H_LPS_DC_DN	-0.47	-2.14	2.73E-09	0
GSE37533_PPARG1_FOXP3_VS_PPARG2_FOXP3_TRANSDUCED_CD4_TCELL_PIOGLITAZONE_TREATED_DN	-0.47	-2.15	3.22E-09	0
GSE2770_TGFB_AND_IL4_ACT_VS_ACT_CD4_TCELL_2H_DN	-0.46	-2.13	3.26E-09	0
GSE40685_TREG_VS_FOXP3_KO_TREG_PRECURSOR_UP	-0.48	-2.17	5.93E-09	0
GSE42021_TREG_VS_TCONV_PLN_UP	-0.46	-2.1	8.25E-09	0
GSE42021_TREG_PLN_VS_CD24INT_TREG_THYMUS_DN	-0.46	-2.08	1.05E-08	0
GSE13485_DAY3_VS_DAY7_YF17D_VACCINE_PBMC_DN	-0.49	-2.18	1.11E-08	0
GSE13485_CTRL_VS_DAY3_YF17D_VACCINE_PBMC_DN	-0.47	-2.13	1.21E-08	0
GSE1432_CTRL_VS_IFNG_6H_MICROGLIA_DN	-0.46	-2.1	1.47E-08	0
GSE36527_CD69_NEG_VS_POS_TREG_CD62L_LOS_KLRG1_NEG_UP	-0.47	-2.14	1.91E-08	0
GSE42021_TREG_PLN_VS_CD24LO_TREG_THYMUS_DN	-0.47	-2.11	2.44E-08	0

GSE18791_CTRL_VS_NEWCASTLE_VIRUS_DC_6H_DN	-0.44	-2.02	4.23E-08	0
GSE37533_PPARG2_FOXP3_VS_FOXP3_TRANSDUCED_CD4_TCELL_DN	-0.45	-2.05	4.70E-08	0
GSE21927_SPLEEN_C57BL6_VS_4T1_TUMOR_BALBC_MONOCYTES_DN	-0.45	-2.03	5.02E-08	0
GSE17974_CTRL_VS_ACT_IL4_AND_ANTI_IL12_24H_CD4_TCELL_DN	-0.44	-2.03	6.00E-08	0
GSE19888_ADENOSINE_A3R_INH_PRETREAT_AND_ACT_BY_A3R_VS_TCELL_MEMBRANES_ACT_MAST_CELL_UP	-0.47	-2.11	7.34E-08	0
GSE13485_DAY1_VS_DAY21_YF17D_VACCINE_PBMC_DN	-0.44	-2.02	8.72E-08	0
GSE21546_WT_VS_SAP1A_KO_DP_THYMOCYTES_UP	-0.46	-2.08	1.21E-07	0
GSE26890_CXCR1_NEG_VS_POS_EFFECTOR_CD8_TCELL_UP	-0.44	-1.99	1.65E-07	0
GSE42021_TREG_PLN_VS_TREG_PRECURSORS_THYMUS_DN	-0.45	-2.01	2.35E-07	0
GSE38681_WT_VS_LYL1_KO_LYMPHOID_PRIMED_MULTIPOTENT_PROGENITOR_DN	-0.44	-2.01	3.05E-07	0
GSE19888_ADENOSINE_A3R_INH_VS_ACT_WITH_INHIBITOR_PRETREATMENT_IN_MAST_CELL_UP	-0.46	-2.05	3.52E-07	0
GSE14000_UNSTIM_VS_4H_LPS_DC_TRANSLATED_RNA_DN	-0.45	-2.03	4.36E-07	1.00E-04
GSE33424_CD161_INT_VS_NEG_CD8_TCELL_UP	-0.43	-1.96	4.33E-07	1.00E-04
GSE22886_UNSTIM_VS_IL15_STIM_NKCELL_DN	-0.41	-1.94	4.66E-07	1.00E-04
GSE40685_TREG_VS_FOXP3_KO_TREG_PRECURSOR_DN	-0.44	-1.99	5.08E-07	1.00E-04
GSE37534_UNTREATED_VS_PIOGLITAZONE_TREATED_CD4_TCELL_PPARG1_AND_FOXP3_TRASDUCED_DN	-0.42	-1.94	5.05E-07	1.00E-04
GSE19888_ADENOSINE_A3R_INH_VS_TCELL_MEMBRANES_ACT_MAST_CELL_UP	-0.44	-1.98	5.35E-07	1.00E-04
GSE10325_BCELL_VS_LUPUS_BCELL_DN	-0.42	-1.94	5.40E-07	1.00E-04
GSE7509_UNSTIM_VS_IFNA_STIM_IMMATURE_DC_DN	-0.46	-2.03	6.75E-07	1.00E-04
GSE37416_CTRL_VS_6H_F_TULARENSIS_LVS_NEUTROPHIL_DN	0.43	2.01	7.37E-07	1.00E-04
GSE22886_NAIVE_CD4_TCELL_VS_48H_ACT_TH1_DN	-0.41	-1.92	7.83E-07	1.00E-04
GSE42088_UNINF_VS_LEISHMANIA_INF_DC_2H_DN	0.43	1.97	8.58E-07	1.00E-04
GSE360_HIGH_DOSE_B_MALAYI_VS_M_TUBERCULOSIS_DC_DN	-0.43	-1.95	8.70E-07	1.00E-04
GSE13485_DAY1_VS_DAY3_YF17D_VACCINE_PBMC_DN	-0.42	-1.93	8.68E-07	1.00E-04
GSE21360_NAIVE_VS_QUATERNARY_MEMORY_CD8_TCELL_UP	-0.41	-1.89	9.41E-07	1.00E-04
GSE18791_CTRL_VS_NEWCASTLE_VIRUS_DC_8H_DN	-0.44	-2	1.01E-06	1.00E-04
