# Supplementary material for: Air pollution exposure is associated with gene expression in children
Source: Environ Epigenet. 2024 Dec 21;10(1):dvae025. doi: 10.1093/eep/dvae025 (PMC11668970; doi:10.1093/eep/dvae025)
Supplement: dvae025_Supp [file dvae025_supp.zip › suppl_data/Supplementary Table 5.pdf]

Supplementary Table 5: Immunologic gene sets overlapping in GSEA analysis of Generation R and ALSPAC

ID_GenR	NES_GenR	pvalue_GenR	p.adjust_GenR	NES_ALSPAC	pvalue_ALSPAC	p.adjust_ALSPAC
GSE13485_DAY1_VS_DAY7_YF17D_VACCINE_PBMC_DN	-2.0248731	3.52E-09	8.57E-06	-2.543357	7.74E-16	3.77E-12
GSE13485_DAY3_VS_DAY7_YF17D_VACCINE_PBMC_DN	-1.9645064	5.19E-08	8.43E-05	-2.1759059	1.11E-08	2.71E-06
GSE18791_UNSTIM_VS_NEWCATSLE_VIRUS_DC_18H_DN	-2.0043987	8.14E-08	9.92E-05	-1.9089356	8.14E-06	0.00050873
GSE21360_NAIVE_VS_QUATERNARY_MEMORY_CD8_TCELL_UP	-1.8164803	2.12E-06	0.00206575	-1.8866102	9.41E-07	9.35E-05
GSE18791_CTRL_VS_NEWCASTLE_VIRUS_DC_10H_DN	-1.7982004	6.69E-06	0.00496848	-1.7589125	5.52E-05	0.00222404
GSE14000_UNSTIM_VS_4H_LPS_DC_DN	-1.8132167	9.36E-06	0.00506418	-2.1416402	2.73E-09	9.50E-07
GSE42724_NAIVE_BCELL_VS_PLASMABLAST_UP	-1.7709587	9.21E-06	0.00506418	-2.2619994	2.40E-10	1.16E-07
GSE19888_ADENOSINE_A3R_INH_VS_ACT_WITH_INHIBITOR_PRETREATMENT_IN_MAST_CELL_UP	-1.825969	1.79E-05	0.00728725	-2.0503469	3.52E-07	4.90E-05
GSE14000_UNSTIM_VS_4H_LPS_DC_TRANSLATED_RNA_DN	-1.7821941	1.72E-05	0.00728725	-2.0322853	4.36E-07	5.74E-05
GSE21360_NAIVE_VS_QUATERNARY_MEMORY_CD8_TCELL_DN	-1.7816204	1.59E-05	0.00728725	-1.8329998	9.25E-06	0.0005632
GSE37533_PPARG1_FOXP3_VS_FOXP3_TRANSDUCED_CD4_TCELL_DN	-1.7688141	2.17E-05	0.00813309	-2.2282569	1.23E-10	9.73E-08
GSE2770_TGFB_AND_IL4_ACT_VS_ACT_CD4_TCELL_2H_DN	-1.7482848	5.24E-05	0.01688395	-2.1255872	3.26E-09	9.93E-07
GSE42021_TREG_VS_TCONV_PLN_UP	-1.7124323	8.64E-05	0.02273659	-2.1000766	8.25E-09	2.23E-06
GSE18791_UNSTIM_VS_NEWCATSLE_VIRUS_DC_10H_DN	-1.6949703	9.33E-05	0.02273659	-2.2658981	2.11E-10	1.14E-07
GSE18791_CTRL_VS_NEWCASTLE_VIRUS_DC_6H_DN	-1.6948587	7.96E-05	0.02273659	-2.0221008	4.23E-08	8.25E-06
GSE40685_TREG_VS_FOXP3_KO_TREG_PRECURSOR_UP	-1.6577029	0.00013139	0.02560606	-2.1703944	5.93E-09	1.70E-06
GSE21546_WT_VS_SAP1A_KO_DP_THYMOCYTES_UP	-1.6574428	0.00013753	0.02577095	-2.0837691	1.21E-07	1.89E-05
GSE10325_CD4_TCELL_VS_LUPUS_CD4_TCELL_DN	-1.6530843	0.00015825	0.0272905	-2.3775565	3.37E-12	5.47E-09
GSE13485_DAY1_VS_DAY3_YF17D_VACCINE_PBMC_DN	-1.6207672	0.0002502	0.03482756	-1.9290247	8.68E-07	8.83E-05
GSE18791_CTRL_VS_NEWCASTLE_VIRUS_DC_8H_DN	-1.6500512	0.00034096	0.04259329	-2.0010746	1.01E-06	9.85E-05
